# Detection and identification of pathogenic trypanosome species in tsetse flies along the Comoé River in Côte d’Ivoire

**DOI:** 10.1051/parasite/2015018

**Published:** 2015-06-02

**Authors:** Vincent Djohan, Dramane Kaba, Jean-Baptiste Rayaissé, Guiguigbaza-Kossigan Dayo, Bamoro Coulibaly, Ernest Salou, Fabien Dofini, Alain De Marie Koffi Kouadio, Hervé Menan, Philippe Solano

**Affiliations:** 1 Institut Pierre Richet/INSP 01 BP 1500 Bouaké Côte d’Ivoire; 2 Université Félix Houphouët Boigny BPV 34 Abidjan Côte d’Ivoire; 3 CIRDES BP 454 Bobo-Dioulasso Burkina Faso; 4 IRD UMR 177 INTERTRYP-IRD-CIRAD 34398 Montpellier France

**Keywords:** Trypanosomes, Trypanosomiasis, Riverine tsetse flies, Côte d’Ivoire

## Abstract

In order to identify pathogenic trypanosomes responsible for African trypanosomiasis, and to better understand tsetse-trypanosome relationships, surveys were undertaken in three sites located in different eco-climatic areas in Côte d’Ivoire during the dry and rainy seasons. Tsetse flies were caught during five consecutive days using biconical traps, dissected and microscopically examined looking for trypanosome infection. Samples from infected flies were tested by PCR using specific primers for *Trypanosoma brucei* s.l., *T. congolense* savannah type, *T. congolense* forest type and *T. vivax*. Of 1941 tsetse flies caught including four species, i.e. *Glossina palpalis palpalis*, *G. p. gambiensis*, *G. tachinoides* and *G. medicorum*, 513 (26%) were dissected and 60 (12%) were found positive by microscopy. Up to 41% of the infections were due to *T. congolense* savannah type, 30% to *T. vivax*, 20% to *T. congolense* forest type and 9% due to *T. brucei* s.l. All four trypanosome species and subgroups were identified from *G. tachinoides* and *G. p. palpalis*, while only two were isolated from *G. p. gambiensis* (*T. brucei* s.l., *T. congolense* savannah type) and *G. medicorum* (*T. congolense* forest, savannah types). Mixed infections were found in 25% of cases and all involved *T. congolense* savannah type with another trypanosome species. The simultaneous occurrence of *T. brucei* s.l., and tsetse from the palpalis group may suggest that human trypanosomiasis can still be a constraint in these localities, while high rates of *T. congolense* and *T. vivax* in the area suggest a potential risk of animal trypanosomiasis in livestock along the Comoé River.

## Abstract (fr)

Dans le but d’identifier les trypanosomes responsables de trypanosomoses africaines humaine et animale et de mieux connaître leurs relations avec les tsé-tsé, des enquêtes ont été réalisées dans trois sites situés dans différentes zones éco-climatiques de Côte d’Ivoire et ce, durant les saisons sèche et pluvieuse. Les glossines ont été capturées durant 5 jours consécutifs en utilisant des pièges biconiques, disséquées et examinées au microscope à la recherche d’infection trypanosomienne. Des échantillons de glossines infectées ont été analysées par PCR en utilisant des amorces spécifiques à *Trypanosoma brucei* s.l., *T. congolense* type savane, *T. congolense* type forêt et *T. vivax*. Sur les 1941 tsé-tsé capturées et comprenant 4 espèces, *Glossina palpalis palpalis*, *G. p. gambiensis*, *G. tachinoides* et *G. medicorum*, 513 (26 %) ont été disséquées et 60 (12 %) ont été trouvées positives par microscopie. Une proportion allant jusqu’à 41 % des infections était due à *T. congolense* type savane, 30 % à *T. vivax*, 20 % à *T. congolense* type forêt et 9 % à *T. brucei* s.l. Les quatre espèces et sous-groupes de trypanosomes ont été isolés chez *G. tachinoides* et *G. p. palpalis* tandis que seulement deux ont été isolés chez *G. p. gambiensis* (*T. brucei* s.l., *T. congolense* type savane) et *G. medicorum* (*T. congolense* types forêt et savane). Les infections mixtes ont été retrouvées dans 25 % des cas et toutes impliquaient *T. congolense* type savane et une autre espèce de trypanosome. La présence simultanée de *T. brucei* s.l. et de mouches tsétsé du groupe palpalis suggère que la trypanosomose humaine peut encore être une contrainte dans ces localités. Le taux élevé de *T. congolense* et de *T. vivax* dans cette zone suggère un risque potentiellement élevé de trypanosomose animale chez le bétail le long du fleuve Comoé.

## Introduction

Human African Trypanosomiasis (HAT), or sleeping sickness, one of the most neglected tropical diseases in the world [23], occurs in the most remote areas of Sub-Saharan Africa, where health systems are often deficient or destabilised by wars. Trypanosomiasis caused by *Trypanosoma brucei gambiense* represents up to 98% of the declared cases [7] and is endemic in a geographically limited area of West and Central Africa [26]. The number of reported cases these two last years was fewer than 8000, but this is certainly underestimated due to incomplete surveillance [31]. Animal African Trypanosomiasis (AAT) is also a major constraint to the development of the livestock sector in Sub-Saharan Africa. The disease induces a decrease of livestock productivity and reduces its density up to 70%. Meat and milk sales are reduced by 50%, calving by 20%, while the calf mortality rate is increased by 20% [15, 29]. Tsetse flies are the main vectors of trypanosomes, protozoan parasites of the genus *Trypanosoma*, pathogens of both HAT and AAT [11]. They are therefore a key factor in trypanosomiasis epidemiology by their central role in trypanosome transmission to vertebrate hosts. Identification of trypanosomes in tsetse flies could be a good indicator for HAT and AAT within an area. Nowadays, parasitological diagnosis is used in field conditions [30] but it is not very sensitive, partly due to the low parasitaemia observed in a natural host infection [19, 24], and polymerase chain reaction (PCR) which is very sensitive and specific, is widely used for trypanosome identification in the laboratory [3, 8]. Detection and identification of pathogenic trypanosomes in tsetse flies along the Comoé River will highlight the areas at risk of human and animal trypanosomiasis. This will help in the use of adapted control methods in this increasingly anthropised habitat, as this part of the country has fertile arable land for agriculture and is suitable for livestock. The main purpose of this study was to identify trypanosomes circulating in tsetse along the Comoé River, and to better understand the relationship between tsetse and trypanosomes in this area.

## Materials and methods

### Study sites

**Figure 1.**
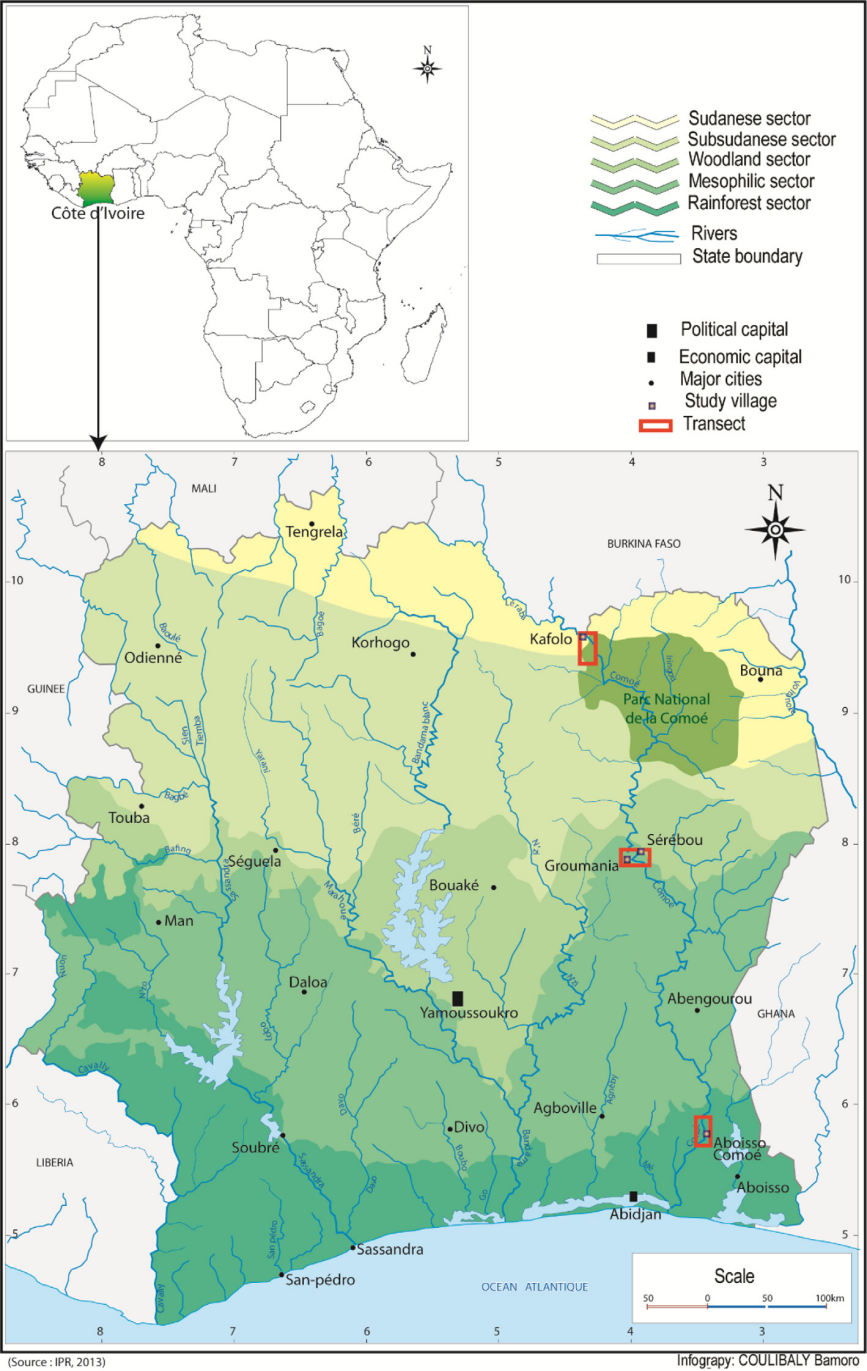
Location of study sites along the Comoé River in Côte d’Ivoire.

Three sites located in different eco-climatic areas were selected to identify pathogenic trypanosomes circulating along the Comoé River in Côte d’Ivoire (Fig. 1). Going from South to North along Comoé River, the first site is located in the South, in Aboisso Comoé, Alépé District (05° 46′ N and 03° 10′ W) in the Yaya Forest Reserve [1]. The vegetation is very lush with dense forest degraded in some places. The second site, between the villages of Groumania and Sérébou (8° 23′ N and 4° 26′ W), lies on the forest-savannah transition area in the middle of the country [1]. The vegetation consists of a mosaic of wet savannah and dry forest along a relatively thin riparian forest. The third site is located in the North, in the Comoé National Park along the border with Burkina Faso near Kafolo village (9° 35′ N and 5° 12′ W) in Kong District, with vegetation consisting of gallery forest along the Comoé River and shrubby savannah hosting some wild game [1].

### Entomological surveys

The surveys were conducted during the dry season (from January 26 to March 10, 2012) and during the rainy season (from October 22 to November 14, 2012) except in Aboisso Comoé where they were only carried out during the dry season. At each site, tsetse were caught using 25 biconical traps [5], set in five radial transects starting from the immediate bank of the river, going to the savannah. Surveys lasted five consecutive days for each season, with a daily collection of catching cages. For each trap, geographic coordinates were recorded using a GPS. Collected tsetse were counted per species and sex and then dissected to isolate on a slide, proboscis, midgut and salivary gland for trypanosome research, using a microscope. When at least one of these organs was found to be infected, all three organs of the tsetse fly were collected individually in Eppendorf^®^ microtubes containing 50 μL of sterile distilled water and kept on the field at about 8° C and then at the laboratory, at −20° C until DNA extraction.

### Identification of trypanosomes

After DNA extraction from the different organs of infected tsetse flies, standard PCR was performed using specific primers for *Trypanosoma congolense* savannah type, *T. congolense* forest type, *T. vivax* west Africa and *T. brucei* s.l. This diagnosis was made using specific satellite sequences of trypanosome taxonomic groups [20, 21]. DNA samples were amplified in 25 μL reaction blend containing: 10 mM Tris-HCl pH 8.3, 50 mM KCl, 200 mM each of four deoxynucleotide triphosphates (dNTPs), 1 μM of each primer and 0.5 units of Taq DNA polymerase. This blend was placed in a thermocycler with the PCR conditions comprising an initial denaturation step at 94° C for 3 min, then 40 cycles of 94° C for 30 s, 55° C for 30 s, and 72° C for 1 min. The elongation step was continued at 72° C for 5 min. Five μL of each amplified sample was resolved by electrophoresis in a 1.5% agarose gel, stained with ethidium bromide and photographed under ultraviolet light. A positive control (with the reference DNA) and a negative control (without DNA and with only distilled water) were added to each reaction series.

### Statistical analysis

Data were analysed using the Statistical Package for the Social Sciences (SPSS) Version 16.0 software. The proportions were statistically analysed with the Chi square test and comparison of means was performed using Student’s *t*-test.

## Results

### Entomological surveys

A total of 1941 tsetse flies were caught on the three sites with 1307 (67.4%) during the dry season and 633 (32.6%) during the rainy season. Caught tsetse fly species and subspecies proportional abundances were 67.1% (1303) for *G. tachinoides*, 16.8% (327) for *G. p. gambiensis*, 15.5% (300) for *G. p. palpalis*, and 0.5% (10) for *G. medicorum*. *G. p. palpalis* was caught only in the southern (Aboisso Comoé) and central (Groumania) parts of the country, while the others (but not *G. p. palpalis*) were caught in the north at the Comoé National Park (Kafolo). Tsetse fly apparent densities per trap (ADP) on the three different sites are presented in Table 1.

**Table 1.** Apparent Density per Trap (ADP) and tsetse infection rate depending on the species, areas and seasons.

Site	Species	Dry season			Rainy season			Stat. (*p*)
		Abundance (*n*, %)	ADP	% Infection	Abundance (*n*, %)	ADP	% Infection	
	*G. tachinoides*	928 (78.3%)	7.424	16.84	375 (82.4%)	3	16.67	0.99
Kafolo	*G. p. gambiensis*	252 (21.3%)	2.016	7.89	75 (16.5%)	0.6	7.14	0.72
	*G. medicorum*	5 (0.4%)	0.04	0	5 (1.1%)	0.04	40	–
Groumania	*G. p. palpalis*	99 (100%)	0.7	1.12	178 (99.4%)	1.432	8.72	0.0081
Aboisso-Comoé	*G. p. palpalis*	23 (100%)	0.184	10	–	–	–	–
Kafolo	*G. tachinoides*	928 (78.3%)	7.424	16.84	375 (82.4%)	3	16.67	
	*G. p. gambiensis*	252 (21.3%)	2.016	7.89%	75 (16.5%)	0.6	7.14	
	Stat. (p)	0.00045			0.0253			

ADP: Apparent Density per Trap.

Sixty (60) flies from a total of 513 dissected were found positive for trypanosome infection using microscopes, yielding an overall infection rate of all species combined of 11.7%. No salivary gland infection was observed. On the other hand, the proboscis and midgut were, respectively, infected at 4.1% and 6.8%. Proboscis and midgut combined infections were observed in 0.8% of cases. The tsetse fly infection rate did not significantly vary in Kafolo for *G. tachinoides* and *G. p. gambiensis* whatever the season (*p* > 0.05) (Table 1). In Groumania on the contrary, the infection rate of *G. p. palpalis* significantly increased from 1.12% to 8.72% (*p* = 0.0081) in the rainy season (Table 1). Comparison between tsetse species in Kafolo showed significantly higher infection rates for *G. tachinoides* than *G. p. gambiensis* during the dry season (*p* = 0.00045) as during the rainy season (*p* = 0.0253) (Table 1).

### Identification of trypanosomes

**Figure 2.**
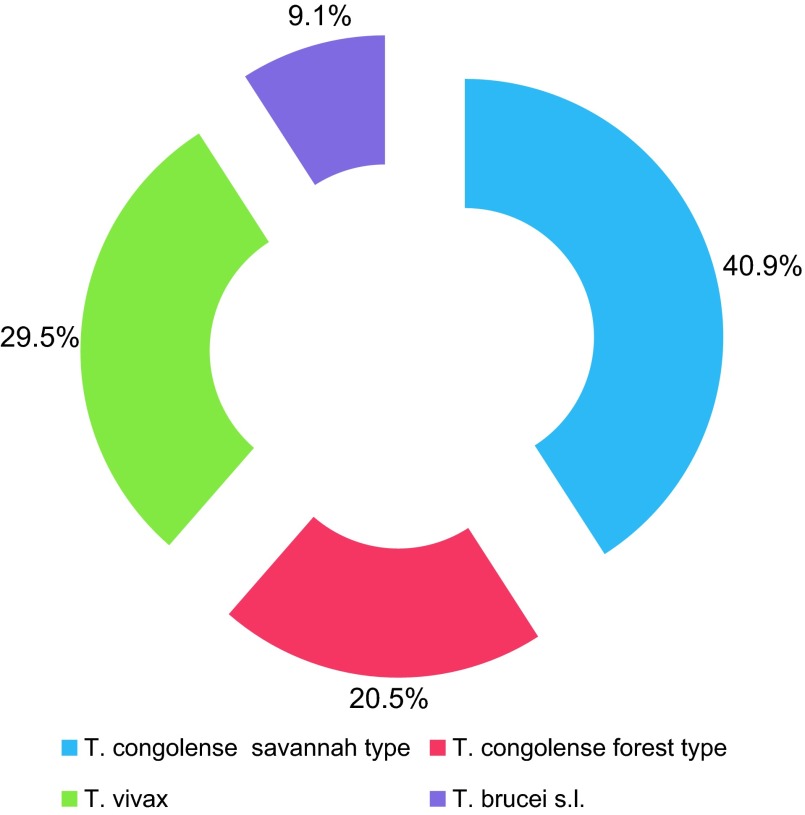
Proportions of trypanosome types circulating along Comoé River in Côte d’Ivoire.

Organs of 58 tsetse flies out of the 60 found positive by microscope were analysed by PCR. A range of 75.9% (44/58) of flies whose samples were analysed by PCR was identified, suggesting that 24.1% of trypanosomes circulating in this area would be some species other than those targeted in this study. From the 44 samples that were identified, 18 (40.9%) were *T. congolense* savannah type, 13 (29.5%) were *T. vivax*, 9 (20.5%) were *T. congolense* forest type and 4 (9.1%) were *T. brucei* s.l. (Fig. 2). *T. congolense* s.l. represents 61.4% of trypanosome species circulating along Comoé River. All types of trypanosomes were identified in *G. tachinoides* and *G. p. palpalis*, unlike *G. p. gambiensis* and *G. medicorum* in which only two were found, namely *T. brucei* s.l. and *T. congolense* savannah type for *G. p. gambiensis* and *T. congolense* forest type and *T. congolense* savannah type for *G. medicorum* (Table 2). Mixed infections accounted for 25% of all infections (Table 3). Among mixed infections, double infections (22.7%) and triple infections (2.3%) were noted. Mixed infections were mostly found in *G. tachinoides*.

**Table 2.** Trypanosome species and subgroup frequency by tsetse species.

Tsetse species	Tv	Tb	Tcf	Tcs	Total
*G. tachinoides*	12/44 (27.2%)	1/44 (2.3%)	4/44 (9.1%)	10/44 (22.7%)	27/44 (61.4%)
*G. p. palpalis*	1/44 (2.3%)	2/44 (4.5%)	4/44 (9.1%)	5/44 (11.4%)	12/44 (27.3%)
*G. p. gambiensis*	0/44 (0%)	1/44 (2.3%)	0/44 (0%)	1/44 (2.3%)	2/44 (4.5%)
*G. medicorum*	0/44 (0%)	0/44 (0%)	1/44 (2.3%)	2/44 (4.5%)	3/44 (6.8%)
Total	13/44 (29.5%)	4/44 (9.1%)	9/44 (20.5%)	18/44 (40.9%)	44/44 (100%)

Tv: *T. vivax*; Tb: *T. brucei* s.l.; Tcf: *T. congolense forest type*; Tcs: *T. congolense* savannah type.

**Table 3.** Frequency of mixed infections of trypanosomes in tsetse.

Trypanosomes in tsetse	Infection profile	Gt	Gpp	Gpg	Gmed	Total	Frequency (%)
1	Tv	9	1	–	–	10	31.2
1	Tb	1	–	1	–	2	6.3
1	Tcf	–	2	–	–	2	6.3
1	Tcs	3	2	1	1	7	21.9
2	Tv + Tcs	3	–	–	–	3	9.4
2	Tcs + Tb	–	1	–	–	1	3.1
2	Tcf + Tcs	4	1	–	1	6	18.7
3	Tcf + Tcs + Tb	–	1	–	–	1	3.1
Total		20	8	2	2	32	100

Gt: *G. tachinoides*, Gpg: *G. p. gambiensis*, Gpp: *G. p. palpalis*, Gmed: *G. medicorum*, Tv: *T. vivax*, Tb: *T. brucei* s.l., Tcf: *T. congolense* forest type, Tcs: *T. congolense* savannah type.

## Discussion

To improve our knowledge on the distribution of trypanosomes responsible for HAT and AAT in Côte d’Ivoire, tsetse flies were caught and dissected along the Comoé River. The identification of pathogenic trypanosomes in tsetse flies has helped to highlight potential risks for human and animal trypanosomiasis associated with these biotopes. *G. palpalis* and *G. tachinoides* were the two predominant species on these sites, justifying their primary role in trypanosome transmission in Côte d’Ivoire [16]. *G. p. palpalis* was caught in the south (Aboisso-Comoé) and in the centre (Groumania) while *G. p. gambiensis* was only caught in the north (Kafolo). In a previous study undertaken at the far south of the Comoé National Park at Gansé, along Comoé River, Kaba [13] caught, contrarily to our survey, more *G. p. gambiensis* than *G. tachinoides*. The relatively high ADPs in Kafolo may be explained by a steady presence of hosts in this area. This faunal stability would also be the reason why the infection rate does not vary significantly for the two main vectors of this site (*G. tachinoides* and *G. p. gambiensis*) whatever the season. On the same site, *G. tachinoides* was significantly more infested than *G. p. gambiensis* whatever the season (*p* = 0.00045 in the dry season and *p* = 0.0253 in the rainy season). In Groumania, the infection rate of *G. p. palpalis* increased significantly during the rainy season. A combination of factors including favourable climatic conditions for the survival and dispersal ability of this species and abundant vegetation attracting animal hosts [9, 25] may explain this increased infection rate. The absence of infected salivary glands among all the dissected tsetse observed in the present study confirms that the natural infection rate of tsetse salivary glands by trypanosomes of *brucei* complex remains very low [10, 15].

The three targeted trypanosome species in our study, namely *T. vivax*, *T. brucei* and *T. congolense*, which are major parasites of human and animal trypanosomiasis in Africa were identified in this area. Specific primers were used to identify the exact type and species of trypanosomes circulating in this area. For *T. vivax*, specific primers for the West Africa type were used. However, we did not use specific primers for subtypes of *T. brucei*, *T. simiae* and *T. congolense* Kilifi, which could partly explain the high rate of 24.1% of non-identified trypanosomes, as well as the presence of other, non-pathogenic trypanosomes not tested in this study (reptilian trypanosomes for instance) [18]. From the pathogenic trypanosomes identified, trypanosomes of cattle are the most encountered along Comoé River including *T. congolense* and *T. vivax*. *T. congolense* accounted for 61.4% of the identified trypanosomes circulating in this area. *T. congolense* savannah type accounted for 41% of infections in contrast to studies in HAT foci in the forest areas of Côte d’Ivoire [12, 20, 21] and Cameroon [22], where *T. congolense* forest type was predominant. A significantly higher infection rate of *T. congolense* savannah type was obtained in the Malanga HAT focus (savannah area) in the Democratic Republic of Congo [27]. This shows that *T. congolense* savannah type would be best suited to animal hosts living in savannah, while *T. congolense* forest type would be suited for animals living in the forest area. However, the extensive use of PCR and DNA probes on naturally infected tsetse has shown that “savannah” trypanosomes may be found in “forest” tsetse [20–22], as well as the reverse, i.e., “forest” trypanosomes in “savannah” tsetse [17]. Also, a significant association between the savannah and forest type of *T. congolense* in tsetse of the *palpalis* and *morsitans* groups has been demonstrated by Solano et al. [28]. The presence of the two species, *T. congolense* and *T. vivax*, known for their pathogenicity to cattle is an indicator of the magnitude of AAT in this area, especially near the Comoé National Park (north) and in Groumania (centre). This suggests that livestock development along this important river will be seriously hampered by the nagana if control measures against tsetse flies and trypanosomes are not taken prior to cattle introduction. The low prevalence of *T. brucei* in tsetse along Comoé River here contrasts with the high prevalence found in HAT foci in Cameroon [22].

Individually, tsetse flies have been variously infected by trypanosomes. *G. medicorum* was infected only by subtypes of *T. congolense*, which certainly reflects its preference for wild ungulates as a feeding source. *T. brucei* s.l. was encountered in *G. p. palpalis*, *G. p. gambiensis* and *G. tachinoides*. *G. p. gambiensis* was infected by *T. brucei* and *T. congolense* forest type. All species of trypanosomes were found in *G. p. palpalis* and *G. tachinoides*, proving that they are the main vectors of human and animal trypanosomiasis in West Africa [2, 4, 14]. *T. congolense* savannah type was found in all species of tsetse fly, an observation also reported in Tanzania [18]. The presence of *T. brucei* along the Comoé River must draw our attention because of the proximity of Abengourou, a former HAT focus [6]. With internal population movements in Côte d’Ivoire due to the socio-political crisis, it cannot be excluded that sleeping sickness patients from areas at risk of HAT, for instance the central-west [6, 14] could move to areas where major vectors and possibly the parasite are present. Therefore, surveillance of HAT should take this into account. Mixed infections that represent a quarter of the infections are in a relatively large proportion and above all increase the risk of AAT. Indeed, *T. congolense* savannah type is present in all combinations of multiple infections. Mixed infections were also observed in the central-west HAT foci in Côte d’Ivoire including Sinfra and Daloa [12, 20].

The trypanosomiasis risk is usually related to tsetse flies density, trypanosome infection rates, and contact between host and vectors [31]. Along the Comoé River, these factors are present in varying degrees. In this area, tsetse flies found are among the major vectors of HAT and AAT in Côte d’Ivoire and the trypanosome infection rate is relatively high. This study showed that pathogenic trypanosomes and the major vectors of HAT and AAT are present along the Comoé River. It is therefore necessary to take these data into account before developing activities such as livestock rearing.
